# Effects of Rumen-Protected β-Alanine on Hepatic Gluconeogenesis-Related Indices and Muscle Energy Metabolism in Transported Cattle

**DOI:** 10.3390/ani16193074

**Published:** 2026-09-29

**Authors:** Boya Fu, Yitian Zang, Haoxiang Xu, Daci Fu, Guwei Lu, Kang Mao, Qinghua Qiu, Xianghui Zhao, Mingren Qu, Kehui Ouyang, Yanjiao Li

**Affiliations:** 1 Jiangxi Province Key Laboratory of Animal Nutrition, Animal Nutrition and Feed Safety Innovation Team, College of Animal Science and Technology, Jiangxi Agricultural University, Nanchang 330045, China; 2 College of Life Science, Nanchang Normal University, Nanchang 330032, China

**Keywords:** bulls, meat quality, muscle glycogen, rumen-protected β-alanine, transport stress

## Abstract

Preslaughter nutritional strategies that preserve energy reserves in transported beef cattle may help maintain normal post-mortem muscle metabolism and meat quality. β-alanine, a carnosine precursor, enhances glucose metabolism and glycogen reserves. This study evaluated the effects of rumen-protected β-alanine (RP-β-Ala) on meat quality, hepatic gluconeogenesis-related indices, and muscle energy metabolism in long-distance transported cattle. Thirty-six healthy 18-month-old Simmental crossbred bulls (627 ± 41 kg) were randomly assigned diets containing 0 (control) or 96 g/head/d RP-β-Ala. After 28 days of feeding, serum was sampled from six bulls per group. Following a 2-day rest (RP-β-Ala supplementation continued), these twelve bulls were transported, slaughtered, and sampled. Transport significantly increased serum lactate dehydrogenase (LDH) and creatine kinase (CK). The transport × RP-β-Ala interaction significantly affected serum superoxide dismutase (SOD). Post-slaughter tissue indices were analyzed solely for the effect of RP-β-Ala supplementation, as all slaughtered bulls underwent transportation. Compared to controls, the RP-β-Ala group exhibited significantly higher muscle pH_45 min_ and an accelerated pH decline rate within 24 h post-mortem in both *M. longissimus dorsi* (LD) and *M. semitendinosus* (ST) muscles. In LD muscle, hardness, shear force, gumminess, and chewiness significantly decreased, while ATP content and HK, PFK, PK, and LDH activities significantly increased. In ST muscle, lactate content and GP, HK, PFK, and LDH activities significantly increased. In the liver, glucose, lactate, ATP, and ADP contents significantly increased, alongside enhanced G-6-Pase and pyruvate carboxylase (PC) activities. Dietary RP-β-Ala supplementation may regulate energy metabolism in transported cattle by enhancing muscle glycolytic activity and indices associated with hepatic gluconeogenic capacity, thereby improving selected meat-quality traits.

## Simple Summary

Long-distance transport causes stress in beef cattle and can lead to dark, firm, and dry (DFD) meat, reducing its commercial value. This study tested whether feeding rumen-protected β-alanine, a compound involved in energy metabolism, could improve meat quality and energy status in transported beef bulls. Thirty-six bulls were fed either a control diet or a diet supplemented with rumen-protected β-alanine for 28 days before transport and slaughter. The results showed that supplementation improved several texture-related parameters of the *M. longissimus dorsi* (LD) muscle, including hardness and shear force, and altered enzyme activities related to energy metabolism in both muscle and liver. However, it did not affect other meat-quality traits such as color, drip loss, or cooking loss. These results suggest that rumen-protected β-alanine may provide selective benefits for meat quality in transported bulls, though further studies are needed to confirm these effects.

## 1. Introduction

Dark, firm, and dry (DFD) beef is a major quality defect in the bovine meat market, and higher incidences of DFD beef proportionally contribute to economic losses [1]. Pre-slaughter transport is the main cause of DFD beef [2]. Long-distance transport exposes cattle to continuous stressors including vibration, crowding, fasting, water deprivation, and thermal extremes [3], which deplete muscle glycogen reserves, thereby increasing the risk of DFD beef [4]. With insufficient glycogen, less lactate and H^+^ are produced post-mortem, leading to a higher ultimate pH (pH_u_) and the development of DFD beef. DFD meat is characterized by undesirable sensory traits and reduced microbial inhibition, making it more prone to spoilage—marked by off-flavors and green discoloration.

Energy depletion represents a critical factor contributing to transport stress and the subsequent development of DFD beef [1]. Consequently, pre-slaughter nutritional management has emerged as an effective technical strategy. Specifically, providing appropriate nutritional supplementation before slaughter may help regulate energy metabolism, preserve muscle glycogen, and support normal post-mortem acidification in transported cattle [1]. β-alanine is a recognized precursor for carnosine synthesis, a compound naturally abundant in meat [5]. Supplementation with β-alanine reliably raises muscle carnosine content [6]. Carnosine serves as a natural energy booster and a science-backed ergogenic aid with documented anti-fatigue effects [7]. Critically, carnosine enhances glucose metabolism, increasing liver and muscle glycogen content [8,9]. Furthermore, β-alanine improves meat quality by enhancing muscle fiber characteristics (density, tenderness), reducing shear force, mitigating oxidative stress, and limiting cellular damage [5,10,11,12,13].

Hu et al. [14] reported that supplementation with unprotected β-alanine improved rumen microbial crude protein (MCP) supply and increase the digestibility of dry matter, organic matter, and crude protein, as well as N retention in beef steers. Subsequently, Zhang et al. [15] reported that supplementation with rumen-protected β-alanine (RP-β-Ala) improved apparent nitrogen retention and nitrogen utilization efficiency. However, no studies have reported on the effects of β-alanine on energy metabolism and meat quality in beef cattle, and it remains unclear whether β-alanine can attenuate excessive energy expenditure during pre-slaughter transport in beef cattle. We hypothesized that feeding β-alanine may influence meat quality by affecting muscle energy metabolism and hepatic glucose production, processes that are compromised during the transport of beef cattle under stress. Therefore, the present study was designed to evaluate the effects of dietary RP-β-Ala supplementation on meat quality, stress responses, muscle glycolysis, and hepatic gluconeogenesis in Chinese Simmental bulls subjected to pre-slaughter transport.

## 2. Materials and Methods

### 2.1. Animal Treatments and Experimental Diets

A total of thirty-six 18-month-old Chinese Simmental crossbred bulls, with an average body weight of 627 ± 41 kg, were randomly assigned to two RP-β-Ala supplemental levels (0 or 96 g/head/d) added to basal diets. Each group had six replicates (pens), with three bulls in each pen. Growth performance data were analyzed with pen as the experimental unit (*n* = 6 pens/treatment, 18 bulls/treatment). For transport and tissue analyses, the individual bull was used as the experimental unit (*n* = 6 bulls/treatment). β-Ala (purity of 99.9%) was purchased from Shandong Jiaoyang Biotechnology Co., Ltd. (Jining, China). Due to the unique digestive anatomy of ruminants, free β-alanine is extensively degraded by rumen microorganisms, resulting in low intestinal bioavailability. To overcome this limitation, a rumen-protected coating technology was used to produce RP-β-Ala, providing a physical barrier that significantly reduces ruminal degradation and ensures intact delivery to the post-ruminal digestive tract for efficient absorption in the small intestine. The production of fat-encapsulated RP-β-Ala was entrusted to Hangzhou King Techina Feed Co., Ltd. (Hangzhou, China), with a coating rate of 50%, a 12 h ruminal bypass ratio of 86.3% as determined by an rumen biomimetic digestion method, and a 12 h intestinal fluid dissolution rate of 81.7% as determined by dissolution test method in artificial intestinal fluid. The supplementation level of 96 g product/head/day was selected with reference to a previous study using RP-β-Ala in beef steers [15], while considering differences in product characteristics and animal body weight. RP-β-Ala was top-dressed onto the basal total mixed ration (TMR) in the feed bunks and manually mixed immediately after the twice-daily feeding at 07:00 and 19:00 h to ensure uniform intake, all animals were offered feed and water ad libitum. Fresh feed was provided daily at scheduled times, and each feeding allotment was added to the trough while ensuring that residual feed remained available at all times, thereby allowing unrestricted intake. The ingredient composition and nutrient levels of the basal diet are presented in Table 1. After 28 days of feeding, one bull with a body weight close to the average was selected from each replicate, totaling twelve bulls (six per treatment), for blood sampling and serum analysis.

Following a 2-day rest period (RP-β-Ala supplementation was not interrupted during these days) to recover from the acute stress induced by handling, weighing, and blood collection, thereby excluding the confounding effects of non-transport-related stress. Subsequently, the twelve bulls were transported to the abattoir and slaughtered for tissue and meat quality sampling. The bulls were fed at 7:00 a.m. and loaded for transport at 4:00 p.m. These 12 experimental bulls together with 12 other bulls were transported by the double layer car, length 6.8 m, width 2.5 m, and height 3.9 m (Shaanxi Automobile Holding Group Co., Ltd., Xi’an, China). The 12 bulls were located on the lower level, with an average of 1.42 m^2^ per bull. The transportation process commenced from Pingxiang City, Jiangxi Province at 4:00 p.m. on 2 December 2022, and concluded in Dongguan City, Guangdong Province at 8:00 a.m. on 3 December 2022, resulting in a total transport duration of 16 h at an average speed of 60 to 70 km/h. The animals were transported for approximately 16 h in a single continuous journey without prolonged stops. Bulls were deprived of water and feed during transportation. The driver made brief stops at service areas along the route, during which accompanying personnel conducted routine checks on animal welfare and vehicle ventilation, and recorded the ambient temperature and relative humidity inside the vehicle. No handling, feeding, or watering was performed during these stops, and the animals remained on the truck throughout the entire journey. During transport, the temperature inside the vehicle fluctuated between 10 °C and 21 °C, while relative humidity ranged from 28% to 56%.

### 2.2. Sample Collection

On the 28th day of the experiment, 10 mL of blood was taken from the tail vein of each bull into evacuated coagulation tubes. The blood samples were centrifuged at 3000× *g* for 10 min at 4 °C to obtain serum samples, which were temporarily stored at −20 °C during the field collection period and subsequently transferred to −80 °C for long-term storage prior to analysis. Upon arrival at the slaughterhouse, the animals were unloaded individually using a covered lairage ramp and held in covered lairage pens (approximately 3 m^2^ per animal) with ad libitum access to water for a 4-h rest period prior to slaughter. This lairage rest period was implemented in accordance with standard commercial and research slaughter protocols to allow partial physiological recovery from transport stress. No feed was provided during lairage. Tail vein blood was collected again to obtain post-transport serum samples. Following a 4 h rest, the bulls were moved individually along a single-file race to the stunning box and rendered unconscious by electrical stunning (applied to the head using captive-bolt-free electrical tongs at 220 V for 3 s). Immediately after stunning, the bulls were shackled, hoisted, and exsanguinated by severing the carotid arteries and jugular veins. Liver samples, *M. longissimus dorsi* (LD) muscle, and *M. semitendinosus* (ST) muscle were taken and stored at −80 °C for analysis of hepatic gluconeogenesis-related indices and muscle energy metabolism. The LD muscle samples were taken from the region between the 12th and 13th ribs of the left half carcass, and the ST muscle samples were taken from the rear of the left half carcass. Steaks of approximately 5 cm in thickness were cut from the LD and ST muscles, with extra-muscular fat and connective tissue removed, from the left half-carcass within 30 min after slaughter. After measuring muscle pH at 45 min (pH_45_ min), these steaks were vacuum-packed and stored at 4 °C for subsequent measurement of muscle pH at 24 h (pH_24_ h), color, drip loss, and texture profile analysis.

### 2.3. Serum Parameters Determination

The concentrations of glucose (catalog number: A154-1-1) and malondialdehyde (MDA, catalog number: A003-1-2), as well as the activities of creatine kinase (CK, catalog number: A032-1-1), lactate dehydrogenase (LDH, catalog number: A020-2), and superoxide dismutase (SOD, catalog number: A001-3-1), were measured and calculated using kits from the Nanjing Jiancheng Bioengineering Institute (Nanjing, China), according to the manufacturer’s instructions.

### 2.4. Meat Quality Measurements

Muscle pH_45 min_ was determined using an insertion-type portable pH meter (HI99163N, Hanna, Padova, Italy). Measurements were performed in triplicate at different areas of each steak and averaged to calculate the results. After muscle pH_45 min_ determination, these steaks were vacuum-packed at 4 °C to measure muscle pH_24 h_, color, drip loss, and texture profile analysis.

Meat color was determined using a spectrocolorimeter (WSC-S, Shanghai, China) at 45 min and 24 h postmortem. The meat color was presented in (CIE) L* (lightness), a* (redness), and b* (yellowness). The illuminant and observer angle were D65 and 10°, respectively. The L*, a*, and b* values were collected from three different areas of each steak on a freshly cut surface exposed to air for 20 min.

To measure drip loss, a muscle sample of approximately 5 cm × 3 cm × 1 cm was excised along the direction of the muscle fibers. After removal of fascia and fat, the initial weight of the sample was measured. The sample was then suspended in a plastic bag without contact with the bag’s inner surface and stored in a refrigerator at 4 °C. After 24 h, the sample was reweighed to calculate the drip loss.

For cooking loss determination, approximately 30 g of muscle was excised using the same sampling method as for drip loss. Following removal of fascia and fat, the initial weight of the meat sample was measured. The meat sample was placed in a plastic bag, from which the air was evacuated, and the bag was sealed to ensure that the meat surface adhered tightly to the plastic. The sealed bag was then immersed in a water bath at 75 °C for 30 min, until the internal center of the meat reached 70 °C. After cooling to room temperature, the surface moisture was blotted with filter paper, and the final weight was measured to calculate the cooking loss.

Texture profile analysis (TPA) of cooked samples was performed using a CT3 texture analyzer (Brookfield, Middleboro, MA, USA). Prior to analysis, surface fat was trimmed from the muscle samples, and samples were excised along the muscle fiber direction and cut into pieces approximately 2.5 cm thick. Each piece was individually vacuum-packaged and immersed in a hot-water bath (80 °C) until the internal center temperature reached 75 °C. The packaged samples were subsequently cooled to room temperature under running water, removed from the bags, and blotted dry with absorbent paper. The samples were further cut into 1.0 cm thick slices to provide a flat surface. For TPA measurement, the slices were placed under the probe with the muscle fibers oriented horizontally, so that the compression and shearing directions were perpendicular to the fiber orientation. Five TPA parameters—hardness, shear force, springiness, gumminess, and chewiness—were then determined. Each sample was tested in triplicate. The instrument settings were as follows: the probe was TA3/100, pre-test speed was 2 mm/s, test speed was 0.5 mm/s, post-test speed was 0.5 mm/s, sample height was 10 mm, and trigger force was 5 g.

### 2.5. Muscle Glycolytic Potential and Glycolytic Enzyme Activities

The glycogen was hydrolysed to glucose by reaction with amyloglucosidase, the glucose-6-phosphate was converted to glucose by reaction with glucose-6-phosphatase [16]. Prior to determination of glucose and lactate concentrations, samples were pretreated by taking 0.1 g of frozen LD muscle or ST muscle samples and homogenizing them in 0.9 mL of ice-cold saline at 4 °C for 6 min, and then the samples were centrifuged in a freezing centrifuge at 2500 r/min for 10 min at 4 °C to obtain the supernatant. The protein concentration in the muscle tissue homogenate was quantified using the BCA Protein Assay Kit (Beijing Alltech Gold Bio Co., Ltd., Beijing, China, catalog number: DQ111). The levels of glucose and lactate were then determined using a commercial kit (Nanjing Jiancheng Bioengineering Institute) according to the manufacturer’s instructions. The glycolytic potential (GP) was calculated according to the formula of Monin and Sellier [17]: GP = 2 × glycogen + lactate.

For the determination of muscle glycolytic enzyme activity, which also requires sample preparation, 0.1 g of frozen muscle sample was homogenized in 0.9 mL of ice-cold saline for 6 min at 4 °C and then centrifuged in a freezing centrifuge at 2500× rpm for 10 min at 4 °C to obtain a supernatant of 10% tissue homogenate. The tissue homogenate was used to determine the activities of pyruvate kinase (PK), creatine kinase (CK), and LDH. Samples used to measure the activities of phosphofructokinase (PFK) and hexokinase (HK) were pre-treated differently by replacing saline with the specific extracts in the kit according to the kit instructions and changing the centrifugation speed to 8000× *g* during centrifugation. The activities of PFK, HK, PK, CK and LDH were determined by the commercially available PFK (catalog number: A129-1-1), HK (catalog number: A077-3), PK (catalog number: A076-1-1), CK (catalog number: A032-1-1), and LDH (catalog number: A020-2) kits (Nanjing Jiancheng Bioengineering Institute). The enzyme activities in muscle were normalized to the total protein concentration in muscle according to the kit instructions.

### 2.6. Muscle Phosphocreatine, Creatine, and Adenosine Phosphates Determination

The phosphocreatine (PCr), creatine, and adenosine phosphates (ATP, ADP, and AMP) contents of LD and ST muscle were assayed using a high-performance liquid chromatography (HPLC) according to a previous method [18]. The reference standard compounds used in the analysis were creatine anhydrous (98%), creatine phosphate disodium salt (97%), adenosine 5’-triphosphate disodium salt, adenosine 5’-diphosphate, adenosine 5’-monophosphate. All of the standard samples were purchased from Shanghai Yuan Mu Biotechnology Co., Ltd. (Shanghai, China). The extracted muscle sample solution was prepared as previously described [18] and analyzed on a Waters e2695 HPLC (Milford, MA, USA) equipped with an Ultimate AQ-C18 column (250 mm × 4.6 mm I.D., 5 μm, Welch Materials, Inc., Shanghai, China) at 25 °C for PCr and creatine determination, and at 30 °C for ATP, ADP, and AMP determination. For the determination of creatine and PCr, the mobile phase consisted of HPLC-grade acetonitrile and a phosphate buffer (29.4 mM potassium dihydrogen orthophosphate and 1.15 mM tetra-butylammonium hydrogen sulphate) in a 2:98 (*v*/*v*) ratio. For the determination of adenosine phosphates, the mobile phase comprised HPLC-grade methanol and a phosphate buffer (2.5 mM tetra-butylammonium hydrogen sulphate, 0.06 M dipotassium hydrogen orthophosphate, 0.04 M potassium dihydrogen orthophosphate) in a 13.5:86.5 (*v*/*v*) ratio. The UV detection for creatine and PCr was at 210 nm, and adenosine phosphates was at 254 nm. The flow-rate was set to 1.0 mL/min and an auto-sequence injection was set for sample measurement. Peaks were identified and quantified using standard curves.

### 2.7. Liver Glucose, Glycogen, and Pyruvate Concentration, and Enzyme Activity Determination

Hepatic glucose, glycogen, and pyruvate concentrations were determined by commercial glucose (catalog number: A154-1-1), glycogen (catalog number: A043-1-1), and pyruvate (catalog number: A076-1-1) kits (Nanjing Jiancheng Bioengineering Institute) according to the manufacturer’s instructions. For liver enzyme activities determination, 0.1 g of frozen liver sample was homogenised in 0.9 mL of ice-cold saline at 4 °C for 6 min, and then centrifuged (3000 r, 10 min, 4 °C) to get the supernatant. The supernatants were used to determine the activities of phosphoenolpyruvate carboxykinase (PEPCK), glucose-6-phosphatase (G-6-pase), and pyruvate carboxylase (PC). The concentration of total protein in liver homogenate was determined by BCA Protein Assay Kit (catalog number: DQ111) (Beijing Alltech Gold Bio Co., Ltd.) according to kit instructions. The activities of PEPCK, G-6-pase, and PC were determined by commercial PEPCK (catalog number: A131-1-1) kit (Nanjing Jiancheng Bioengineering Institute), commercial G-6-pase (catalog number: YJ063687) and PC kits (catalog number: YJ063780) (Shanghai enzyme linked Biotechnology Co., Ltd., Shanghai, China). The enzyme activities in the liver were normalized by total protein concentration in the liver according to the kit instructions.

### 2.8. Statistical Analysis

Data were processed using SPSS (version 26.0, IBM, Armonk, NY, USA). For growth performance, the pen was used as the experimental unit (*n* = 6 pens/treatment). For serum parameters and all post-slaughter tissue and meat quality measurements, the individual bull was used as the experimental unit (*n* = 6 bulls/treatment). Serum-related indicators were analyzed using a repeated-measures mixed model in SPSS. Treatment, transport time point, and their interaction were included as fixed effects, with individual animal fitted as a random effect to account for repeated observations. Data are expressed as means and standard errors of the mean (SEMs). The remaining data were analyzed using an independent-samples *t*-test, with results also expressed as means and SEM. Statistical significance was set at *p* < 0.05.

## 3. Results

### 3.1. Serum Parameters

As shown in Table 2, with the exception of SOD activity, the interaction between RP-β-Ala supplementation and transport did not significantly affect the other serum parameters. The SOD activity in the RP-β-Ala group post-transport was significantly higher than that in the other groups (*p* < 0.05). Furthermore, dietary RP-β-Ala supplementation notably enhanced overall SOD activity compared with the unsupplemented groups (63.45 vs. 56.98 U/mL, *p* < 0.05). Additionally, significant increases in the serum LDH activity (3143.25 vs. 1206.62 U/L) and CK activity (1019.26 vs. 76.62 U/L) were observed post-transport compared to pre-transport (*p* < 0.01).

### 3.2. Meat Quality

**Figure 1:**
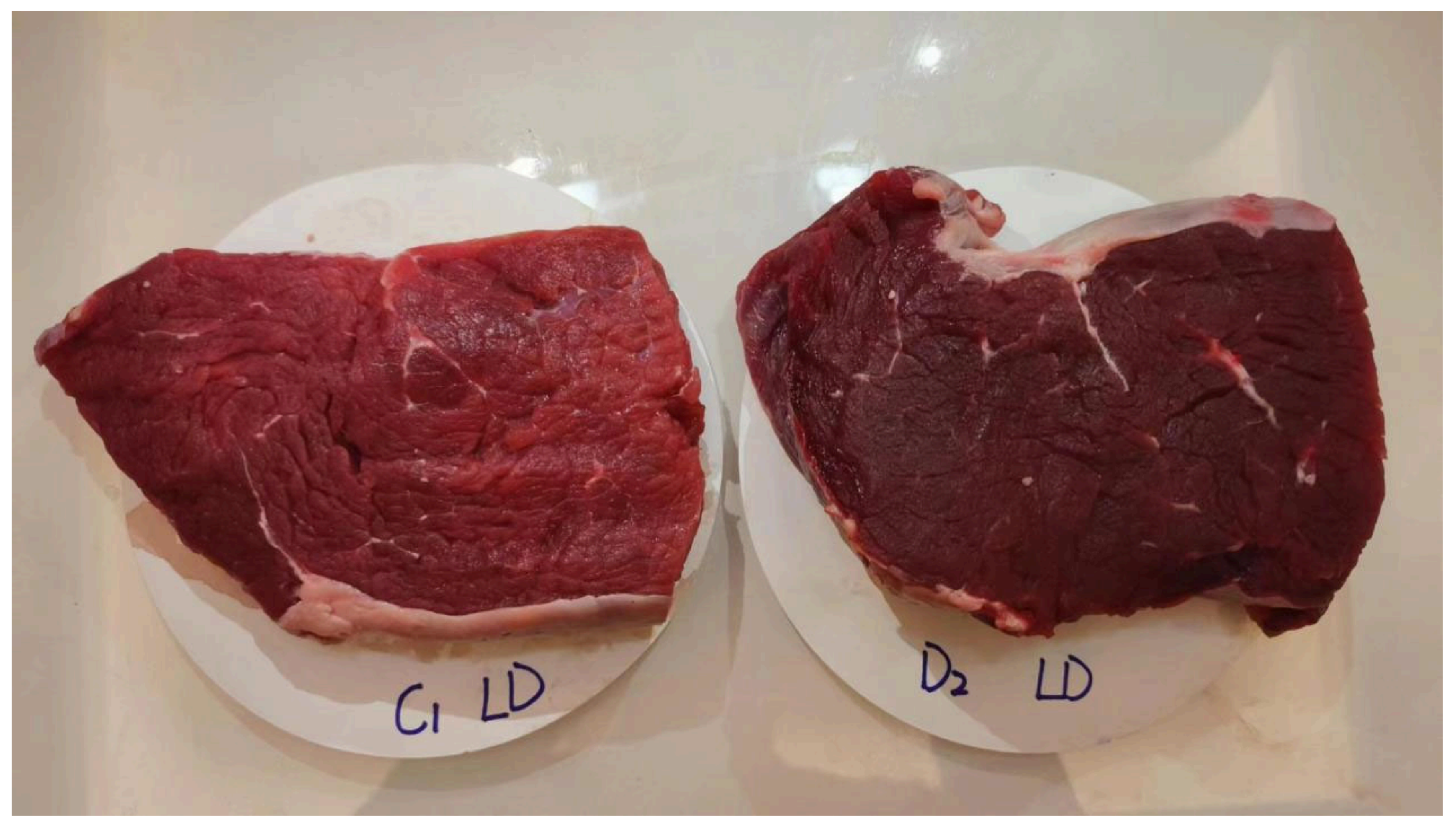
Illustrative images of LD muscle at 24 h post-slaughter. The left image shows a representative RP-β-Ala group sample, whereas the right image shows the sole control sample exhibiting a DFD-like appearance, which was not representative of the control treatment.

At 24 h post-slaughter, one LD sample from the control group exhibited a DFD-like appearance (Figure 1). This isolated observation is descriptive only and does not indicate a treatment difference in DFD incidence. Mean pH_24 h_ did not differ significantly between treatments in either LD or ST muscle (*p* > 0.05).

The effects of dietary supplementation with RP-β-Ala on the quality of LD and ST muscles in beef bulls are presented in Table 3. Compared with the control group, the RP-β-Ala group exhibited a significant increase in muscle pH at 45 min (*p* < 0.05); the magnitude of pH decline within the first 24 h post-mortem was also markedly greater in both the LD and ST muscles. However, the addition of RP-β-Ala did not significantly affect the meat color at 24 h post-slaughter in either the LD or ST muscles (*p* > 0.05). Additionally, the hardness, shear force, and gumminess of the LD muscle in the RP-β-Ala group were significantly lower than those observed in the control group (*p* < 0.05), while chewiness was also significantly lower in the RP-β-Ala group compared to the control group (*p* < 0.01).

### 3.3. Muscle Glycolytic Potential and Glycolytic Enzyme Activity

As shown in Table 4, the RP-β-Ala group showed a trend of increased lactate content in the LD muscle compared to the control group (*p* = 0.089), along with significantly higher lactate content and GP in the ST muscle (*p* < 0.05), representing 75.46% and 69.17%, respectively. In the RP-β-Ala group, the activities of the glycolytic rate-limiting enzymes HK, PFK, PK, and LDH in the LD muscle were significantly higher than those in the control group (*p* < 0.05). Similarly, in the ST muscle of the RP-β-Ala group, the activities of HK, PFK, and LDH were also significantly higher than those in the control group (*p* < 0.05).

### 3.4. Adenosine Phosphates, Phosphocreatine and Creatine in Muscle

As shown in Table 5, in LD muscle, dietary supplemented with RP-β-Ala significantly increased the ATP content, and decreased the AMP content compared to the control group (*p* < 0.05). The ATP/ADP ratio did not show a significant difference between the two groups (*p* > 0.05), while the AMP/ATP ratio in the RP-β-Ala group was significantly lower than that in the control group (*p* < 0.01). In comparison to the control group, the ATP, ADP, and AMP concentrations, along with their ratios in the ST muscle of the RP-β-Ala group exhibited similar trends to those observed in the LD muscle, although the differences were not statistically significant (*p* > 0.05).

### 3.5. Hepatic Energy Metabolism

Table 6 summarizes hepatic metabolites and gluconeogenic enzyme activities. Compared to the control group, RP-β-Ala supplementation significantly increased hepatic lactate content (*p* < 0.05). Additionally, it notably enhanced the activities of the hepatic gluconeogenic rate-limiting enzymes G-6-Pase and PC (*p* < 0.05), with increases of 45.45% and 55.9%, respectively. Furthermore, there was a trend towards an increase in PEPCK activity (*p* = 0.071). The hepatic glucose concentration in the RP-β-Ala group was significantly higher than that in the control group (*p* < 0.05).

As shown in Table 7, RP-β-Ala supplementation increased hepatic ATP and ADP concentrations by 49.4% (*p* = 0.039) and 9.7% (*p* = 0.002), respectively, whereas AMP showed a non-significant upward trend (*p* = 0.139). Consequently, the ATP/ADP ratio tended to increase (*p* = 0.074), whereas the AMP/ATP ratio showed a corresponding downward tendency (*p* = 0.051). Hepatic creatine content showed a similar pattern, rising by 54.6% (*p* = 0.089) relative to the control group.

## 4. Discussion

The post-transport increase in serum CK and LDH activities is a well-established indicator of transport-elicited muscle injury, reflecting fatigue, local hypoxia, and reduced perfusion of skeletal muscle [19]. Previous studies on the long-distance transportation of beef cattle from our group have demonstrated that, following more than 12 h of transportation, these animals experience transport stress-induced oxidative damage [20]. This damage leads to changes in cell membrane permeability and the leakage of stress marker enzymes such as CK and LDH into the serum [21]. In this study, the serum activities of CK and LDH of bulls in both groups significantly increased post-transport compared to pre-transport, indicating that long-distance transport-induced stress responses resulted in increased cell membrane permeability and oxidative cellular damage. β-Alanine has been shown to enhance oxidative metabolic capacity and reduce mitochondrial oxidative stress by-products [22]. As a precursor of carnosine, β-Alanine can alleviate oxidative damage by promoting carnosine synthesis and increasing the activities of SOD and glutathione peroxidase (GSH-Px) in the skeletal muscles of exercising animals. Consistently, in the present study, the serum activity of SOD in the RP-β-Ala supplemented group was significantly higher than that in the control group. Blood glucose concentration is a key indicator of energy metabolism and stress status. During long-distance transportation (greater than 500 km), blood glucose levels tend to decrease as the duration of fasting and energy consumption increases [23,24]. A reduction in blood glucose signifies a negative energy balance, which, if prolonged, can heighten disease susceptibility and exacerbate inflammation and oxidative stress [25]. In our study, serum glucose level in the control group numerically decreased post-transport compared with pre-transport. Conversely, the RP-β-Ala group showed numerically higher serum glucose level after transport compared with the control group. A study in mice demonstrated that combining carbohydrates, alanine, and proline supplementation can effectively maintain blood glucose levels during prolonged exercise and enhance endurance performance [26], indicating that alanine not only regulates blood glucose under resting conditions but also helps maintain stable blood glucose levels through additional energy support during physical activity.

It is widely recognized that pre-slaughter stress is one of the primary causes of inferior meat quality. During long-distance transportation prior to slaughter, chronic stressors such as fasting and fatigue cause excessive depletion of muscle glycogen, insufficient lactate production post-slaughter, elevated ultimate pH, and ultimately the development of DFD meat, characterized by a dark color, firm texture, and dry surface [27,28]. In this study, the pH_45 min_ values of LD and ST muscles in the RP-β-Ala group were significantly higher than those in the control group. We speculate that this difference may be attributed to the capacity of β-alanine to increase carnosine content and to buffer H^+^ ions to some extent [29]. DFD meat is generally identified using ultimate pH together with color and other quality characteristics, although the specific pH threshold varies among countries and classification systems [29,30]. In the present study, RP-β-Ala supplementation significantly increased pH_45 min_ and the magnitude of the pH decline from 45 min to 24 h post-mortem in both the LD and ST muscles. However, mean pH_24 h_ did not differ significantly between treatments in either muscle, and no significant treatment differences were detected in instrumental color measurements. Although one LD sample from the control group showed a DFD-like visual appearance, this isolated observation cannot be interpreted as evidence that RP-β-Ala supplementation reduced DFD incidence. Instead, the present findings support an effect of RP-β-Ala on early post-mortem pH development and selected meat-quality characteristics. A larger study with DFD incidence specified as a primary outcome and assessed using predefined diagnostic criteria is required to determine whether RP-β-Ala affects the occurrence of DFD meat. Texture indicators such as hardness, shear force, gumminess, and chewiness are closely related to muscle tenderness. In this study, the hardness, shear force, gumminess, and chewiness of LD muscle in the RP-β-Ala group were significantly lower than those in the control group, with chewiness exhibiting a highly significant difference. In contrast, no significant differences were observed for any of these indicators in the ST muscle. These results indicate that RP-β-Ala significantly improved tenderness related parameters in the LD muscle, whereas similar changes were not detected in the ST muscle. The differential responsiveness between the two muscle types may be related to their distinct physiological functions and energy metabolism [31]. Of note, the post-mortem pH and meat colour of the ST muscle were superior to those of the LD muscle, which may help explain why the treatment affected LD muscle quality parameters but not those of the ST muscle.

Previous studies have demonstrated that β-alanine mitigates impairments in cellular energy metabolism through the regulation of glycolytic processes [32]. Muscle glycogen content (reflected by GP) determines post-mortem lactate production capacity, while glycolytic enzyme activity controls the rate of glycolysis. Higher glycogen availability, coupled with greater glycolytic enzyme activity, may promote post-mortem lactate accumulation and thereby influence the extent of post-mortem pH decline [18,33]. Bulls subjected to long-distance transportation experience high levels of stress, leading to intense muscle contraction, heightened energy metabolism, and exacerbated glycogen breakdown in muscles, resulting in insufficient muscle energy reserves post-slaughter [34]. Oxidative damage caused by transport stress may also impair glycolytic enzyme activity, inhibit post-slaughter muscle glycolysis, and interfere with normal post-mortem acidification [35]. In this study, compared to the control group, RP-β-Ala supplementation increased the glycogen, lactate, and glucose concentrations in the muscles of bulls following long-distance transportation, significantly enhanced GP in the ST, and markedly increased the activities of key glycolytic enzymes such as HK, PFK, PK, and LDH in both muscle types compared to the control group. This suggests that β-alanine may enhance glycogen reserves in bulls and mitigate the transport-induced decline in muscle glycogen content. Furthermore, the antioxidant capacity conferred by RP-β-Ala in skeletal muscle can help alleviate oxidative damage from transport stress, thereby also attenuating the inactivation of glycolysis-related enzymes. It is now widely accepted that the CK/PCr system serves as the primary energy supply pathway during the initial post-mortem stages of muscle metabolism following animal slaughter. PCr is hydrolyzed to Cr while producing ATP under the action of CK [36]. When approximately 70% of the stored PCr is consumed, the rate of ATP consumption surpasses the rate of ATP production, triggering the initiation of glycolysis to supply energy [37]. In this study, no significant differences were observed in PCr, Cr, and CK levels in the LD and ST muscles of the two groups of bulls compared to the control group; however, the ATP concentration in the LD muscle of the RP-β-Ala group was significantly elevated. We speculate that RP-β-Ala does not affect the CK/PCr system, and the observed increase in ATP concentration in the LD muscle may be attributed to ATP produced via the glycolytic pathway. Therefore, the effect of RP-β-Ala on muscle energy metabolism appears to be linked to its influence on muscle glycolysis. Nevertheless, these biochemical changes should not be interpreted as direct evidence of a reduction in DFD incidence in the present study. However, given the limited sample size (*n* = 6/treatment), the absence of significant differences in ST muscle should not be interpreted as evidence of a true absence of effect, as insufficient statistical power cannot be excluded.

Ruminants, including beef cattle, rely largely on hepatic gluconeogenesis to maintain blood glucose concentrations, with this pathway providing approximately 80% of endogenous glucose supply [38]. Lactate and pyruvate can be transported to the liver and utilized as gluconeogenesis substrates [39]. Gluconeogenic capacity is influenced by substrate availability and hepatic energy status, and may be favored by an increased ATP/ADP ratio [40]. PC, PEPCK, and G-6-Pase are key enzymes in this pathway; however, their activities indicate enzymatic capacity rather than gluconeogenic flux. Alanine is also an important hepatic glucogenic precursor because it can be converted to pyruvate for glucose synthesis. In this study, RP-β-Ala supplementation increased hepatic lactate and glucose contents and the activities of PC and G-6-Pase. These changes are consistent with increased substrate availability and hepatic gluconeogenic capacity. Nevertheless, because gluconeogenic flux and net hepatic glucose output were not directly measured, the present results do not demonstrate increased hepatic glucose production. Further studies using isotope-tracer approaches are required to confirm this possibility.

The differential responses of glucose concentrations in the liver and muscle observed in this study are likely attributable to their distinct metabolic functions. The liver serves as the principal site for gluconeogenesis and glucose release, and the elevated hepatic glucose concentration may reflect an increased gluconeogenic flux (supported by the higher activities of G-6-Pase and PC) that contributes to systemic glucose supply. In skeletal muscle, however, glucose uptake is predominantly mediated by insulin-stimulated GLUT4 translocation and is rapidly directed toward glycolysis or glycogen storage rather than intracellular accumulation. Consequently, an increase in hepatic glucose does not necessarily lead to a parallel increase in muscle glucose, as these two tissues are governed by different regulatory mechanisms [41]. This tissue-specific metabolic partitioning may account for the lack of correlation between hepatic and muscular glucose levels in the present study. Post-slaughter tissue analyses were limited to *n* = 6 bulls/treatment, which restricts statistical power for detecting small-to-medium effect sizes. These findings should therefore be interpreted with caution, and replication in larger studies is warranted.

## 5. Conclusions

This study demonstrates that dietary RP-β-Ala supplementation may alleviate transport stress in beef cattle, as evidenced by increased serum SOD, greater post-slaughter pH decline, and improved LD muscle tenderness. RP-β-Ala also enhanced muscle glycolytic enzyme activities and ST muscle glycolytic potential, while increasing indices associated with hepatic gluconeogenic capacity. However, hepatic gluconeogenic flux was not directly measured. Moreover, the single DFD-like observation and nonsignificant pH_24 h_ difference do not support a reduction in DFD incidence.

## Figures and Tables

**Table 1:** Ingredient composition and nutrient concentration of the basal diet (air-dry basis, %).

Ingredients	Content %
Brewers dried grain	29.74
Straw silage	25.95
Corn	21.84
Soybean meal	6.38
Wheat straw	5.74
Microbial agent	4.73
Wheat bran	2.95
Premix ^1^	1.97
Sodium bicarbonate	0.7
Nutrient concentration	Content %
Dry matter	63.97
Crude protein	14.35
Crude fat	4.68
Crude ash	8.39
Neutral detergent fiber	32.65
Acid detergent fiber	13.23

Note: ^1^ The premix supplied the following nutrients per kilogram of premix: 80,000 IU of Vitamin A, 20,000 IU of Vitamin D_3_, 320 IU of Vitamin E, 3100 mg of Fe, 1500 mg of Mn, 2000 mg of Zn, 650 mg of Cu, 15 mg of Co, 40 mg of I, 115 g of Ca and 35 g of P.

**Table 2:** Effect of RP-β-Ala supplementation on blood metabolites of Chinese Simmental bulls transported for 16 h prior to slaughter.

Items	Treatment ^1^				SEM	*p*-Value		
	Pre-Transport		Post-Transport					
	Control	RP-β-Ala	Control	RP-β-Ala		RP-β-Ala	Transport	Transport × RP-β-Ala ^2^
Glucose (mmol/L)	5.30	5.39	4.71	5.85	0.63	0.299	0.913	0.362
LDH (U/L)	1173.39	1239.86	3189.28	3097.22	244.41	0.891	<0.001	0.362
CK (U/L)	90.25	62.99	1055.55	982.97	416.65	0.699	<0.001	0.861
MDA (nmol/mL)	2.90	3.45	2.72	3.21	0.93	0.087	0.528	0.929
SOD (U/mL)	58.66	59.59	55.30	67.32	7.85	0.022	0.285	0.017

Note: ^1^ Using a 2 × 2 two-factor experimental design, rumen-protected β-Alanine (RP-β-Ala) supplemented to the basal at 0 or 96 g/d per bull. ^2^ Transport × RP-β-Ala, the interaction between Transport and RP-β-Ala. SEM, standard error of the mean; LDH, lactate dehydrogenase; CK, creatine kinase; MDA, malonaldehyde; SOD, superoxide dismutase.

**Table 3:** Effect of RP-β-Ala supplementation on *M. longissimus dorsi* and *M. semitendinosus* muscle quality of Chinese Simmental bulls transported for 16 h prior to slaughter.

Items	Treatment ^1^		SEM	*p*-Value
	Control	RP-β-Ala		
*M. longissimus dorsi*				
pH_45 min_	6.79	7.16	0.09	0.039
pH_24 h_	5.96	5.89	0.05	0.576
pH_45 min_–pH_24 h_	0.84	1.26	0.08	0.004
Lightness (L*)	26.71	28.13	0.65	0.316
Redness (a*)	8.76	9.48	0.26	0.184
Yellowness (b*)	4.19	4.91	0.24	0.139
Drip loss (%)	1.36	1.75	0.38	0.334
Cooking loss (%)	22.65	22.26	5.28	0.943
Hardness (kgf)	12.42	8.43	1.04	0.045
Shear force (N)	121.81	82.71	10.15	0.045
Springiness (mm)	2.40	2.48	0.09	0.675
Gumminess (kg)	1.95	0.78	0.30	0.046
Chewiness (mJ)	88.43	18.75	13.50	0.003
*M. semitendinosus*				
pH_45 min_	6.44	6.97	0.13	0.044
pH_24 h_	5.80	5.70	0.05	0.377
pH_45 min_–pH_24 h_	0.65	1.28	0.15	0.036
Lightness (L*)	29.25	30.63	0.60	0.286
Redness (a*)	9.61	12.43	0.64	0.200
Yellowness (b*)	5.41	6.10	0.44	0.474
Drip loss (%)	1.01	1.46	0.21	0.068
Cooking loss (%)	31.59	32.35	2.14	0.732
Hardness (kgf)	9.40	8.68	0.71	0.646
Shear force (N)	92.20	88.51	7.42	0.823
Springiness (mm)	2.63	2.60	0.08	0.873
Gumminess (kg)	1.52	1.52	0.14	0.977
Chewiness (mJ)	56.95	55.68	6.02	0.925

Note: ^1^ Rumen-protected β-Alanine (RP-β-Ala) supplemented to the basal at 0 or 96 g/d per bull. SEM, standard error of the mean.

**Table 4:** Effect of RP-β-Ala supplementation on the glycolytic potential and glycolytic enzymes activity in *M. longissimus dorsi* and *M. semitendinosus* muscle of Chinese Simmental bulls transported for 16 h prior to slaughter.

Items	Treatment ^1^		SEM	*p*-Value
	Control	RP-β-Ala		
*M. longissimus dorsi*				
Glycogen (μmol/g)	41.87	62.88	7.53	0.196
Lactate (μmol/g)	516.60	685.32	48.41	0.089
GP (μmol/g)	600.53	811.08	62.43	0.103
HK (U/μgprot)	162.22	305.83	33.49	0.024
PFK (U/mgprot)	81.64	115.62	8.00	0.021
PK (U/kgprot)	2.32	2.81	0.12	0.031
LDH (U/kgprot)	3.41	3.85	0.12	0.047
*M. semitendinosus*				
Glycogen (μmol/g)	36.62	46.61	3.58	0.186
Lactate (μmol/g)	488.37	856.87	86.12	0.025
GP (μmol/g)	561.61	950.09	90.63	0.024
HK (U/μgprot)	312.69	793.98	91.61	<0.001
PFK (U/mgprot)	75.44	123.84	12.07	0.040
PK (U/kgprot)	2.13	2.35	0.08	0.215
LDH (U/kgprot)	2.16	3.07	0.22	0.023

Note: ^1^ Rumen-protected β-Alanine (RP-β-Ala) supplemented to the basal at 0 or 96 g/d per bull. SEM, standard error of the mean; GP (glycolytic potential) = 2 × (glycogen) + lactate [17]; LDH, lactate dehydrogenase; PFK, phosphofructokinase; PK, pyruvate kinase; HK, hexokinase.

**Table 5:** Effect of RP-β-Ala supplementation on phosphocreatine, creatine, and adenosine phosphates in *M. longissimus dorsi* and *M. semitendinosus* muscle of Chinese Simmental bulls transported for 16 h prior to slaughter.

Items	Treatment ^1^		SEM	*p*-Value
	Control	RP-β-Ala		
*M. longissimus dorsi*				
ATP (μg/g)	118.83	122.43	0.79	0.014
ADP (μg/g)	415.42	420.71	5.34	0.655
AMP (μg/g)	3095.71	2934.89	108.09	0.009
ATP/ADP	0.29	0.29	0.01	0.483
AMP/ATP	26.05	23.98	0.39	0.001
Cr (μmol/g)	10.08	9.93	0.85	0.935
PCr (μmol/g)	13.01	13.21	0.23	0.698
CK (U/mgprot)	16.29	17.92	0.82	0.358
*M. semitendinosus*				
ATP (μg/g)	112.19	117.58	2.45	0.307
ADP (μg/g)	518.91	528.79	14.03	0.752
AMP (μg/g)	3008.4	2895.66	102.91	0.621
ATP/ADP	0.22	0.22	0.01	0.710
AMP/ATP	26.84	24.48	1.05	0.294
Cr (μmol/g)	21.25	24.09	1.45	0.365
PCr (μmol/g)	13.58	13.01	0.29	0.368
CK (U/mgprot)	26.06	29.11	1.33	0.288

Note: ^1^ Rumen-protected β-Alanine (RP-β-Ala) supplemented to the basal at 0 or 96 g/d per bull. SEM, standard error of the mean; ATP, adenosine triphosphate; ADP, adenosine diphosphate; AMP, adenosine monophosphate; Cr, creatine; PCr, phosphocreatine; CK, creatine kinase.

**Table 6:** Effect of RP-β-Ala supplementation on gluconeogenic substrates and enzyme activities of Chinese Simmental bulls transported for 16 h prior to slaughter.

Items	Treatment ^1^		SEM	*p*-Value
	Control	RP-β-Ala		
Lactate (μmol/g)	253.35	312.48	13.96	0.021
Glycogen (μmol/g)	71.22	80.95	7.98	0.595
Pyruvate (μmol/gprot)	763.61	734.90	27.92	0.645
Glucose (μmol/g)	1.04	1.34	0.07	0.019
G-6-Pase (IU/mgprot)	0.55	0.80	0.06	0.027
PEPCK (U/mgprot)	1932.39	2512.66	159.77	0.071
PC (U/mgprot)	1.36	2.12	0.18	0.026

Note: ^1^ Rumen-protected β-Alanine (RP-β-Ala) supplemented to the basal at 0 or 96 g/d per bull. SEM, standard error of the mean; G-6-pase, glucose-6-phosphatase; PEPCK, phosphoenolpyruvate carboxykinase; PC, pyruvate carboxylase.

**Table 7:** Effect of RP-β-Ala supplementation on phosphocreatine, creatine, and adenosine phosphates in liver of Chinese Simmental bulls transported for 16 h prior to slaughter.

Items	Treatment ^1^		SEM	*p*-Value
	Control	RP-β-Ala		
ATP (μg/g)	107.42	160.53	13.21	0.039
ADP (μg/g)	111.56	122.42	2.09	0.002
AMP (μg/g)	61.01	63.75	0.89	0.139
ATP/ADP	0.96	1.31	0.10	0.074
AMP/ATP	0.60	0.42	0.05	0.051
Creatine (μmol/g)	17.96	27.77	2.83	0.089
PCr (μmol/g)	0.83	0.85	0.08	0.876

Note: ^1^ Rumen-protected β-Alanine (RP-β-Ala)supplemented to the basal at 0 or 96 g/d per bull. SEM, standard error of the mean; ATP, adenosine triphosphate; ADP, adenosine diphosphate; AMP, adenosine monophosphate; PCr, phosphocreatine.

## Data Availability

Upon reasonable request, the datasets of this study can be available from the corresponding authors.
